# A multiscale computational framework to evaluate flow alterations during mechanical thrombectomy for treatment of ischaemic stroke

**DOI:** 10.3389/fcvm.2023.1117449

**Published:** 2023-03-15

**Authors:** Ivan Benemerito, Ahmed Mustafa, Ning Wang, Ana Paula Narata, Andrew Narracott, Alberto Marzo

**Affiliations:** ^1^INSIGNEO Institute for in silico Medicine, The University of Sheffield, Sheffield, United Kingdom; ^2^Department of Mechanical Engineering, The University of Sheffield, Sheffield, United Kingdom; ^3^Department of Neuroradiology, University Hospital of Southampton, Southampton, United Kingdom; ^4^Department of Infection, Immunity and Cardiovascular Disease, The University of Sheffield, Sheffield, United Kingdom

**Keywords:** ischaemic stroke, middle cerebral artery, mechanical thrombectomy, balloon guide catheter, stent retriever, aspiration, 1D model, computational

## Abstract

The treatment of ischaemic stroke increasingly relies upon endovascular procedures known as mechanical thrombectomy (MT), which consists in capturing and removing the clot with a catheter-guided stent while at the same time applying external aspiration with the aim of reducing haemodynamic loads during retrieval. However, uniform consensus on procedural parameters such as the use of balloon guide catheters (BGC) to provide proximal flow control, or the position of the aspiration catheter is still lacking. Ultimately the decision is left to the clinician performing the operation, and it is difficult to predict how these treatment options might influence clinical outcome. In this study we present a multiscale computational framework to simulate MT procedures. The developed framework can provide quantitative assessment of clinically relevant quantities such as flow in the retrieval path and can be used to find the optimal procedural parameters that are most likely to result in a favorable clinical outcome. The results show the advantage of using BGC during MT and indicate small differences between positioning the aspiration catheter in proximal or distal locations. The framework has significant potential for future expansions and applications to other surgical treatments.

## 1. Introduction

Ischaemic stroke (IS) might happen when there is a reduction or blockage of cerebral blood flow limiting the supply of oxygen and nutrients to areas of the brain. It occurs when a cerebral vessel becomes obstructed by a blood clot that has become dislodged from another site in the cardiac circulation (emboli or thromboemboli). IS is reported in approximately 87% of all stroke cases (1) and, of these, more than half affect the middle cerebral artery (MCA) (2). Patients with thrombus located in the MCA are the most common target for treatment since this artery is a crucial distributor of blood to large areas of the brain. The Circle of Willis (CoW), a ring of arteries which connects the anterior and posterior brain circulation is responsible for providing collateral blood pathways in case of occlusion of a major vessel. Because of the modifications that it induces on the cardiovascular system, age is widely recognized as one of the most important risk factors associated with IS, with the risk of suffering from it doubling every decade after 55 years of age (3). Some authors have reported localized increase in stiffness in large elastic arteries such as the aorta (4), while others observed global modifications (5). These phenomena affect the wave propagation within the arterial tree, a crucial determinant of the arterial pressure, and ultimately raise the systolic and diastolic pressure over time (5). However, after 55 years of age a reduction in diastolic pressure and a further increase in systolic pressure is typically observed (5). Stiffening also reduces the ability of the distal vasculature to dampen flow pulsatility and this results in increase in peripheral resistance and decrease in peripheral compliance (5). The global increase in vascular resistance, combined with the physiological thickening of the cardiac walls, diminishes the efficacy of the heart in pumping blood into the vascular network and results in a drop of cardiac output (6).

In terms of treatment, the pharmacological dissolution of the clot through intravenous thrombolytic therapy has recently been replaced as the state-the-art treatment by the mechanical retrieval of the clot *via* endovascular treatments, which have shown high success rates of blood flow recanalization and post-treatment survival in a number of randomized clinical trials (7, 8). In these techniques, known as mechanical thrombectomy (MT), a catheter-guided stent is first inserted through a groin puncture and navigated to the occlusion site, and then used to penetrate, capture and remove the clot (9). The vessels crossed by the thrombus as it is removed from the body are referred to as the “retrieval path” or “critical path.” Clot fragmentation can happen during MT (10), generating thousands of fragments that can be carried by the blood flow away from the clot site and potentially occlude other vessels in positions more difficult to treat. The risk of distal embolization is partially mitigated by the use of flow control devices, which reduce the haemodynamic forces acting on the clot by applying suction (11). These devices are being increasingly used in combination with balloon guided catheters (BGCs): a small balloon is inflated in a critical location, typically at the junction between Internal Carotid (ICA), External Carotid and Common Carotid arteries, to temporarily impede flow while a stent retrieval device catches and removes the thrombus, facilitated by the applied suction. This method is particularly effective when large-bore aspiration catheters are used (12) and when their tip is deployed at a short distance from the proximal end of the clot (13). However, the navigation of the aspiration catheter through tortuous vessels such as the internal carotid artery (ICA) is a complex operation (14) and can occasionally result in vascular damage and post-surgery complications (15). There is debate in the literature on how to optimize the technique in these situations (16), namely, where is the best location to apply the suction (10, 15, 17, 18) and what aspiration flow rate guarantees the best outcome.

Computational models offer the possibility to flexibly address these questions, and to embrace the complexity of the treatment and its many variables without endangering patients. To study the CoW, researchers have produced three dimensional representations of the complete network (19) and its most common anatomical variations (20). The treatment of a 3D problem in computational fluid dynamics (CFD) involves the solution of the Navier-Stokes equations. This is already challenging when assuming rigid walls, and its treatment can be further complicated when the fluid dynamic model is coupled to the transient problem of pulse wave propagation in systems with deformable walls (21). The definition of the subject specific 3D computational domain requires the segmentation of medical images, a process that is highly time consuming and can suffer from lack of repeatability. In the last few years, 3D models have been used to simulate clot retrieval in rigid vessels and without the explicit inclusion of the blood (22). The models developed by Luraghi et al. (22) contain a patient specific representation of the anatomy, as well as a detailed mesh of the stenting device. Mechanical properties of the thrombus are defined following experimental characterization of clots produced *in vitro*. Because of their high accuracy, these models are being used as fundamental building blocks for the development of *in silico* clinical trials (23). Recently, the authors included both a clot fragmentation model and external aspiration (24). However, since the fluid phase is not present in their models, the haemodynamic forces caused by aspiration are estimated and implicitly represented through a traction force acting on the proximal part of the clot.

One dimensional models, being relatively inexpensive to produce and run, can be used to flexibly and effectively investigate various scenarios during pre-operative planning and take into account the natural variability that is found in large populations (4). Although limited by their lower-dimensionality representation of the geometry and physics of the problem, they often include a representation of wall mechanics and the interaction between transmural pressure and vessel lumen area, thus allowing the representation of an important phenomenon of the cardiovascular system, that of pulse wave propagation (25, 26). When used to address appropriate research questions, these models can provide answers that are equally as valid as 3D models but in significantly shorter time frames. Researchers have investigated the effect of vessel occlusion on the haemodynamics of the CoW using 1D models: in Refs. (27) and (28) Padmos and colleagues coupled an extensive, steady-state 1D cerebral network to a diffusion model to study how the collateral circulation supports brain perfusion in case of IS; a similar study was performed in Phan et al. (29), where the authors assessed the conditions necessary for distributed collateral circulation to provide perfusion above a 30% threshold; in 2022, Benemerito and co-authors combined the 1D description with statistical emulators to identify a number of easily measurable biomarkers that correlate strongly with distal MCA perfusion following an ischaemic event (30). To the best of our knowledge, only one study has been published that simulates the retrieval of a clot and the consequent impact on the haemodynamic of the CoW (31), where they modeled the movement of the clot as arterial stenosis of variable magnitude. This study, however, does not include an aspiration catheter.

In this work we aim at developing a modeling framework to simulate the aspiration during the thrombectomy procedure using a 1D model of the cerebral circulation. The framework is applied to a virtual population of 65 year old patients with the intention of assessing the efficacy of proximal flow control devices in influencing the cerebral circulation in the MCA. We also aim to assess how, in the case of MT performed with BGC, the location of the aspiration catheter and the applied aspiration flow rate influence flow in the critical path and its neighboring vessels. Finally, we identify the configurations more likely to deliver favorable haemodynamic conditions during surgery.

## 2. Materials and methods

The study is composed of two parts. In the first part, we further developed and validated a 1D cerebral circulation model that simulates blood flow in a virtual population of healthy individuals across a broad age range. In the second part, we applied the modeling approach to simulate a typical MT procedure with proximal flow control, and then investigate the influence of aspiration on reverse flow in the region of interest.

We will first briefly present the main features of the 1D modeling framework we adopted for this study, and then describe how it is used for the simulation of ageing and aspiration thrombectomy.

### 2.1. 1D modeling framework

The 1D modeling framework is based on a previous implementation developed by Melis et al. (32). It is built using the open source software openBF (33), which relies on the finite volume method and the MUSCL scheme (34) to iteratively solve the problem of describing the blood circulation in a network of elastic vessels.

Within the context of 1D modeling, arteries are described as long, straight, narrow tubes which can deform elastically in the radial direction while undergoing zero-displacement in the longitudinal direction. To obtain the equations for 1D systems, the conservation of mass and momentum are expressed in cylindrical coordinates and then integrated along the radial direction. A complete formulation also requires the definition of a constitutive relationship which relates the transmural pressure with the area of the vessel, and an estimate of the shape of the velocity profile. The assumption of a parabolic profile yields the following hyperbolic system of PDEs:


$$\left\{ \begin{array}{c} \frac{\partial A}{\partial t}+\frac{\partial Q}{\partial z}=0\\ \frac{\partial Q}{\partial t}+\frac{\partial }{\partial z}\,\left(\alpha \,\frac{Q^{2}}{A}\right)+\frac{A}{\rho }\,\frac{\partial P}{\partial z}=-2\,\mu \,\left(\gamma_{v}+2\right)\,\frac{Q}{A}\\ P\,\left(A\right)=P_{e\,x\,t}+\beta \,\left(\sqrt{\frac{A}{A_{0}}}-1\right),\beta =\sqrt{\frac{\pi }{A_{0}}}\,\frac{E\,h_{0}}{1-\nu^{2}} \end{array}\right.$$

The system describes the dynamics of a single elastic vessel with cross sectional area *A* and flow rate *Q*. The vessel is characterized by its diastolic lumen area *A*_0_ and its wall thickness *h*_0_. The values *E* and ν represent Young’s modulus and Poisson’s ratio, respectively. The blood is characterized by its density ρ and viscosity μ. The quantity γ_*v*_ describes the shape of the velocity profile while α is the Coriolis coefficient. The first equation represents the conservation of mass, while the second one the conservation of momentum. The third equation is the constitutive relationship between cross sectional area and transmural pressure and is needed to close the problem. The equations are solved for each vessel in the system. Interface conditions are imposed to join the solution from different vessels: at conjunctions, the conservation of mass and total pressure is imposed, while mass and static pressure are preserved at bifurcations and anastomoses. The peripheral vasculature is included in the model through three-element windkessel models consisting of a resistive component in series with the parallel of a one resistive and one capacitive component. At each time step during the solution process, impedance matching is performed to minimize spurious wave reflections (32). More details on interface and boundary conditions can be found in Sherwin et al. (25). The equations are solved by iteratively computing the flow rate, vessel cross-sectional area, blood pressure and blood velocity in all the vessels of the network. The convergence of the method is assessed by evaluating the similarity of the current and previous pressure solution at the mid-point of each vessel. This is done through the L2 norm of their difference.


$$L\,2_{i}^{n}=\sqrt{\int_{t_{0}}^{t_{f}}{\left|P_{i}^{n}-P_{i}^{n-1}\right|}^{2}\,dt},$$

where $P_{i}^{n}$ is the pressure solution at the mid-point of vessel *i* during iteration *n*, and the integral is computed over the cardiac cycle from *t*_0_ to *t*_*f*_. The convergence criterion is applied to all the vessels in the network. The iteration proceeds until the largest error in the entire network falls below a prescribed threshold (3 mmHg). The results presented in this study refer to the last simulated cardiac cycle.

### 2.2. Generation of virtual populations of ageing individuals

**FIGURE 1:**
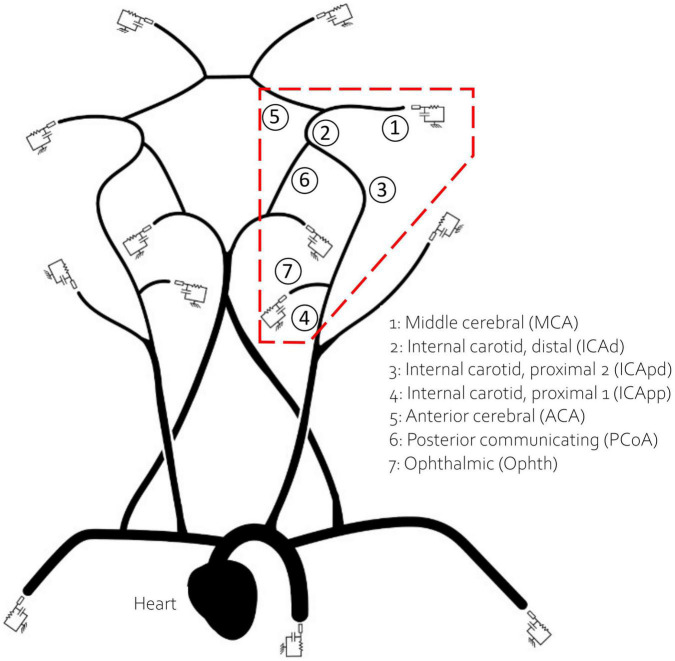
The network used in this study. The inlet boundary condition is applied at the heart. The 13 outlet vessels are coupled to three element windkessel models. Vessels in the red dashed polygon, which encircles the retrieval path, are numbered and named for reference.

1D models available in the literature are typically built using geometrical data from different sources often from young individuals. As previously proposed by Charlton et al. (6), to obtain a virtual population of 65 year olds we artificially aged a reference baseline model by modifying its parameters according to the known physiological changes associated with ageing. The reference model, originally published by Alastruey et al. (35), included the main vessels of the upper systemic circulation such as the ascending aorta and the brachial artery, as well as a representation of the CoW, MCA, distal ACA and PCA. In this study we have also included the Ophthalmic artery, a small vessel that branches from the internal carotid artery (ICA) and usually acts as separation point for positioning the aspiration catheter (36). The network is represented in Figure 1.

A calibration procedure was performed to identify model parameters resulting in pressure waveforms observed in the literature. To this end, we simulated a preliminary virtual population of 1,000 healthy subjects by scaling the network parameters according to the corresponding statistical distribution in Table 1 for age 25. We then identified the subject whose value of systolic and diastolic brachial pressure was in best agreement with the literature. The outcome of this constituted the baseline model at 25 years. Details are provided in Supplementary material.

**TABLE 1:** Distribution of scaling parameters for the ageing model.

Age	Radius		Thickness		Stiffness		Resistance		Compliance		Cardiac output	
	Mean	Std	Mean	Std	Mean	Std	Mean	Std	Mean	Std	Mean	Std
25	1.00	0.06	1.00	0.18	1.00	0.16	1.00	0.22	1.00	0.27	1.00	0.23
35	1.03	0.06	1.10	0.20	1.08	0.16	1.04	0.23	0.88	0.22	0.95	0.22
45	1.04	0.07	1.12	0.22	1.23	0.16	1.10	0.25	0.77	0.21	0.90	0.22
55	1.07	0.07	1.25	0.24	1.29	0.26	1.21	0.27	0.65	0.18	0.86	0.21
65	1.13	0.07	1.52	0.26	1.63	0.35	1.23	0.28	0.54	0.15	0.80	0.20
75	1.16	0.07	1.75	0.28	2.00	0.45	1.46	0.29	0.42	0.12	0.75	0.17

The reported values are expressed as the ratio of the value at a specific age with respect to the baseline population at 25 years.

For the ageing model we described the physiological ageing process in six virtual populations of individuals of different ages (25, 35, 45, 55, 65, and 75). This step was essential to obtain a representative population of 65 year old individuals, for whom the incidence of IS is particularly high. Table 1 summarizes the information available about physiological changes of specific input parameters due to ageing, represented as the mean and standard deviation of the distribution of percentage variations, with respect to the baseline population at 25 years. In a previously published study (6), the authors modeled ageing subjects by modifying the values of arterial radius, thickness, stiffness, peripheral resistance and compliance and cardiac output, and generated 729 individuals per age group. In this study we followed a similar approach and generated 1,000 subjects per age group. To generate a single virtual patient at a particular age we sampled the scaling parameters for radius, thickness, stiffness, peripheral resistance and compliance, and cardiac output, from the normal distributions in Table 1. The scaling parameters were multiplied by the network parameters from the baseline model at 25 years to generate the virtual subjects. Care was taken to ensure that the resulting virtual networks had the same geometrical symmetry properties of the original network. The baseline inlet flow condition, imposed at the ascending aorta, was prescribed as half a sinusoidal wave with peak amplitude of 350 ml/s and duration 0.3 s (systole). During the 0.7 s diastole the inlet flow rate was set to 0 ml/s (35). The inlet waveform was also scaled according to the scaling factor for cardiac outlet. Simulations were run using openBF on the local HPC cluster.

Using the same approach as (6), we filtered out the results from those simulations that showed values of systolic or diastolic brachial pressure outside the physiological range. Virtual patients were identified as outside this range if either their systolic or diastolic brachial pressure fell outside 2.575 standard deviations from the reference mean values measured by McEniery et al. (37) during the Anglo-Cardiff Collaborative Trial (ACCT) and shown in Table 2. Of the 1,000 individuals in the 65 year old population, 211 led to physiological results and were simulated during a MT procedure. Further details on the number of subjects in the physiological group are presented in the section “3. Results”.

**TABLE 2:** Distributions of systolic and diastolic brachial pressure with age.

Age	Systolic pressure (mmHg)		Diastolic pressure (mmHg)	
	Mean	Std	Mean	Std
25	124	10	75	8
35	123	10	77	7
45	125	9	79	6
55	125	9	79	6
65	126	9	78	6
75	127	9	76	6

From McEniery et al. (37).

We also evaluated the average velocity in the MCA in different age groups, as well as the MCA pulsatility index (PI). PI is defined, with obvious notation, as.


$$P\,I=\frac{V_{m\,a\,x}-V_{m\,i\,n}}{V_{m\,e\,a\,n}}$$

Only a few studies have reported measurements of the mean velocity in the MCA in patients of different ages (38–40). Results tend to show low coefficients of linear correlation and therefore we only performed an analysis of the trend shown by the MCA mean velocity and PI predicted by the ageing model.

### 2.3. Simulation of the mechanical thrombectomy

**FIGURE 2:**
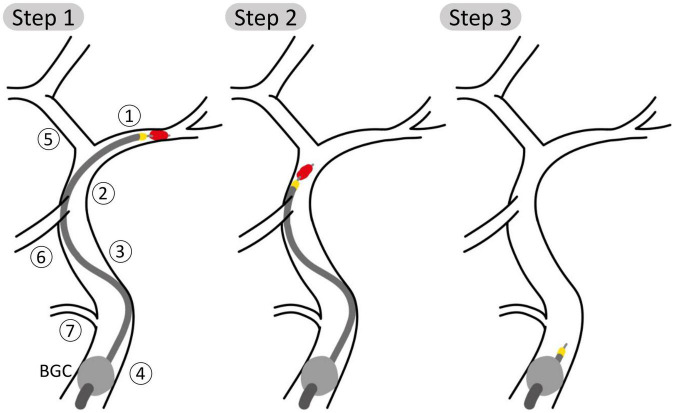
Schematic representation of steps 1, 2, and 3 of the aspiration thrombectomy procedure with the balloon guided catheter (BGC, gray ball) placed in the Internal Carotid, proximal 1 (ICApp) and aspiration applied on the proximal face of the thrombus. Vessel numbering is the same as in Figure 1. **(Step 1)** The aspiration catheter and stent retriever engage the clot; **(Step 2)** the clot is retrieved while aspiration is still applied; **(Step 3)** following successful removal of the clot, aspiration is still applied to avoid distal embolization.

Each of the 211 physiological 65 year old individuals was given an IS in the left MCA. The stroke was described as a complete occlusion of the artery, modeled by imposing zero flow at the corresponding outlet (35). Following this, we performed a simulated MT procedure on each patient. The procedure is depicted in Figure 2 and modeled as follows:

(1) The BGC is inflated at the bifurcation between in the ipsilateral proximal ICA, External Carotid and Common Carotid arteries. The presence of the BGC was modeled by disconnecting the junction between these three vessels and imposing a zero-flow boundary condition at the proximal part of the proximal ICA. The aspiration, included as an additional inlet, is started when the stent retriever is mobilized. We simulated three distinct aspiration locations between proximal and distal ICA (ICApp, ICApd, and ICAd).
(2) Then, for each aspiration location, the retrieval of the clot stent retriever was simulated by subsequently placing the occlusion in the vessels of the retrieval path between the MCA and the aspiration point. The following arteries are part of the retrieval path: middle cerebral (MCA); internal carotid, distal (ICAd); proximal internal carotid, segment distal to the Ophthalmic artery (ICApd); proximal internal carotid, segment proximal to the Ophthalmic artery (ICApp). Following the approach taken by Phan et al. (31), during the retrieval phase the moving clot was modeled as a 70% stenosis of the artery where the clot was located.
(3) Following the removal of the clot, aspiration is still applied to ensure that fragments are not embolized. During this stage, pre-intervention boundary conditions were restored in the MCA.

**FIGURE 3:**
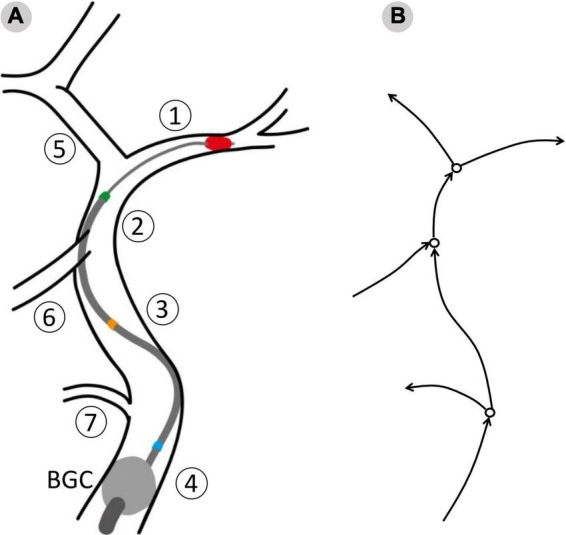
The surgical procedure as modeled in openBF. Vessel numbering is the same as in Figure 1. **(A)** The balloon guided catheter (BGC, gray circle) is located at the junction between Internal, External and Common Carotid. Three positions of the aspiration catheter are shown: Blue, aspiration in ICApp; Orange, aspiration in ICApd; GREEN, aspiration in ICAd. **(B)** 1D representation of the retrieval path and neighboring vessels. Arrows indicate the positive direction of flow.

This process resulted in twelve possible combinations of clot location and position of the tip, or network configurations. To evaluate the effects of the aspiration rate, each patient was simulated in all the possible configurations and using 3 different aspiration rates (0.5, 5, 10 ml/s). The value 5 ml/s is commonly used in clinical practice (11). In total, 7,596 (3 aspiration rates for each of the 12 configurations of 211 physiological patients) MT simulations were run. A summary of the network configurations is presented in Table 3, and a representation of the procedure as modeled in openBF is shown in Figure 3.

**TABLE 3:** Network configurations for simulations of mechanical thrombectomy.

Location of BGC	Location of aspiration	Location of clot	Configuration	MCA outlet	Phase
ICApp	ICApp	MCA	C1	FO	1
		ICAd	C2	PR	2
		ICApd	C3	PR	2
		ICApp	C4	PR	2
		Removed	C5	PR	3
	ICApd	MCA	C6	FO	1
		ICAd	C7	PR	2
		ICApd	C8	PR	2
		Removed	C9	PR	3
	ICAd	MCA	C10	FO	1
		ICAd	C11	PR	2
		Removed	C12	PR	3

MCA, middle cerebral; ICAd, internal carotid, distal; ICApd, internal carotid, proximal 2; ICApp, internal carotid, proximal 1. In the “MCA outlet” column, FO stands for “Full Occlusion” while PR for “Pre-intervention.”

The effects of aspiration rate were evaluated by studying the resulting distributions of mean flow in the MCA, retrieval path and neighboring vessels. The mean flow is defined as the time average of the vessel flow rate over a cardiac cycle.

## 3. Results

### 3.1. Ageing model

**FIGURE 4:**
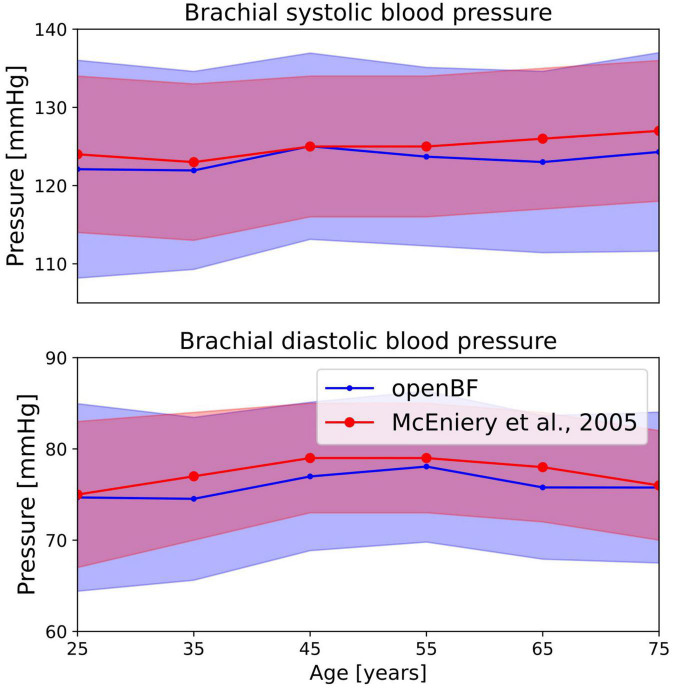
Outcome of the ageing model, comparison of brachial pressure against experimental data from McEniery et al. (37). Simulation results are shown in blue, validation points are in red. Solid line indicates the mean values while shaded areas represent one standard deviation.

A total of 26% of the 6,000 virtual subjects that were generated were physiologically plausible. The mean and standard deviation of brachial systolic and diastolic pressures are shown in Table 4 and represented as line plots in Figure 4 compared with the literature data.

**TABLE 4:** Outcome of the ageing model: Brachial pressure.

Age	# Of subjects	Systolic pressure (mmHg)		Diastolic pressure (mmHg)	
		Mean	Std	Mean	Std
25	280	122.10	13.92	74.68	10.27
35	308	121.94	12.66	74.53	8.92
45	268	125.04	11.91	76.98	8.13
55	275	123.70	11.41	78.07	8.28
65	211	123.01	11.60	75.78	7.87
75	206	124.30	12.69	75.77	8.27

We observe the expected monotonic increase in the systolic pressure from 122 to 124 mmHg, which matches the experimental results. Brachial diastolic pressure increases from 75 mmHg at 25 years to 77 mmHg at 55 years, and then constantly declines until 75 mmHg at 75 years.

**FIGURE 5:**
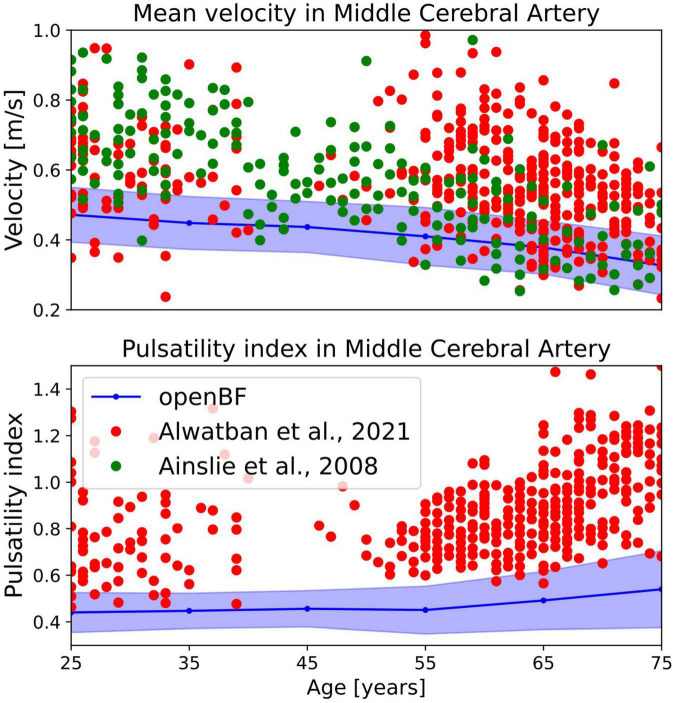
Outcome of the ageing model, comparison of MCA mean velocity (top row) and pulsatility in the MCA (bottom row) against experimental data. Simulation results are shown in blue, validation points from Alwatban et al. (40) are shown in red, while points from Alwatban et al. (39) are shown in green.

In terms of average MCA velocity we observe a monotonic decrease from 0.45 to 0.30 m/s. As shown in Figure 5, our model predicted MCA velocities that are lower than those observed by Alwatban et al. (40) in their study on 524 participants of comparable age, or by Ainslie et al. (39) on 307 subjects. We predicted a steady increase of PI steadily increases in the age range under considerations, from 0.44 to 0.56. As for the MCA velocity, also PI is underpredicted when compared to the experimental data from Alwatban et al. (40).

### 3.2. Thrombectomy procedure

#### 3.2.1. MCA flow

In this and the following section we present the results from the MT simulation on the population of 211, 65 year old virtual patients. The distribution of average MCA flow during the active retrieval phase is shown in Table 5. With 0.5 ml/s aspiration applied, average MCA flows are 130.59 (72.79, 215.68), 134.91 (75.06, 224.25), and 134.02 (75.18, 224.13) ml/min when the aspiration point is located in ICApp, ICApd and ICAd, respectively. Increasing the aspiration to 5 ml/s reduces the flow by 35%, and a further increase yields an additional 50% reduction.

**TABLE 5:** Average values of median MCA flow (ml/min) during the active retrieval phase.

	Location of BGC	Location of aspiration	Aspiration: 0.5 ml/s	Aspiration: 5 ml/s	Aspiration: 10 ml/s
**Median (minimum,** **max) of flow [ml/min]**	ICApp	ICApp	130.59 (72.79, 215.68)	94.73 (47.39, 167.49)	60.6 (19.38, 126.05)
		ICApd	134.91 (75.06, 224.25)	99.98 (53.81, 177.11)	64.8 (21.96, 133.81)
		ICAd	134.02 (75.18, 224.13)	98.17 (53.73, 176.90)	63.29 (20.85, 130.41)

Results are presented for the three aspiration rates. ICApp, internal carotid, proximal 1; ICApd, internal carotid, proximal 2; ICAd, internal carotid, distal. Cells are coloured according to the location of the aspiration catheter, as shown in Figure 3.

**FIGURE 6:**
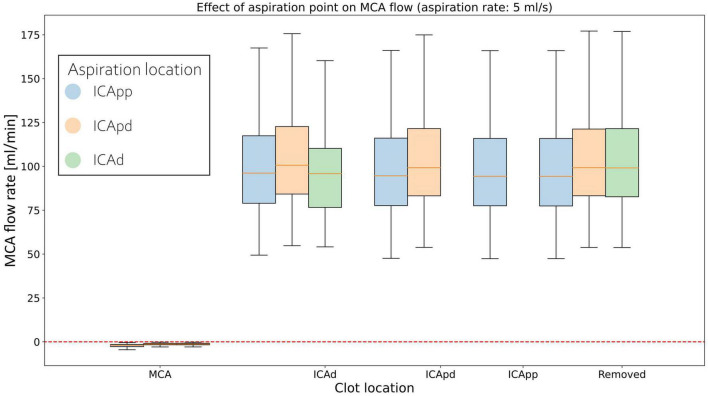
Distributions of average MCA volumetric flow rate during clot retrieval, for three different positions of the aspiration catheter (ICApp, ICApd, and ICAd) when 5 ml/s aspiration is applied. BLUE: Aspiration in the proximal part of the proximal Internal Carotid (ICApp); ORANGE: Aspiration in the distal part of the proximal Internal Carotid (ICApd); GREEN: Aspiration in distal Internal Carotid (ICAd). The number of bars of a specific colour depends on the length of the retrieval path for the corresponding aspiration location (i.e., with aspiration in ICAd the retrieval path consists of MCA, ICAd, ICApd, and ICApd; with aspiration in ICAd the retrieval path consists only of MCA and ICAd).

Middle cerebral artery flows for individual clot locations during retrieval are shown in Figure 6 for 5 ml/s aspiration. At the beginning of the procedure the MCA average flow is practically zero since the clot is fully obstructing the artery. As this is dislodged from its initial location, MCA flow sharply increases to a value that remains essentially constant during the entire retrieval process. This is observed in all twelve network configurations.

#### 3.2.2. Haemodynamic conditions

**FIGURE 7:**
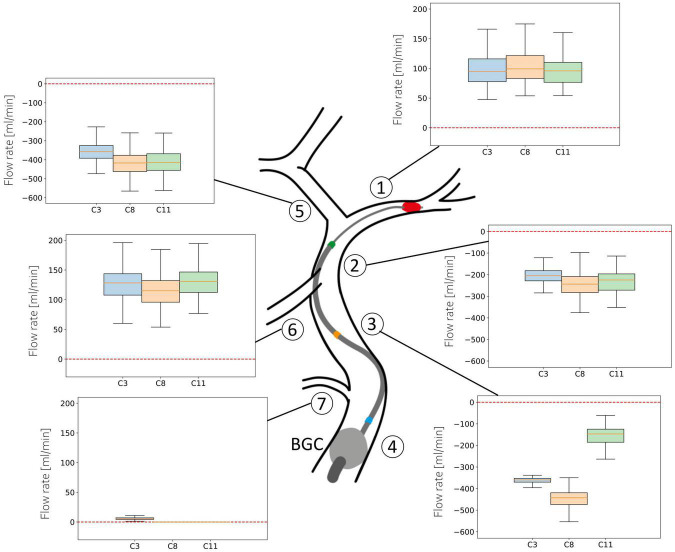
Haemodynamic conditions in the vessels neighboring the retrieval path. Results are shown for the 211 subjects in the 65 year old population with 5 ml/s aspiration rate in configurations C3, C8, and C11. Details of the configurations are given in Table 3.

Figure 7 shows the distribution of average flow along the retrieval path (MCA, distal ICA, proximal ICA after the Ophthalmic artery) and in its neighboring vessels (ACA, PCoA, and Ophth) during the simulated MT, for three different locations of the aspiration catheter (ICApp, ICApd and ICAd) and two different clot locations (ICApd and ICAd). This corresponds to the network configurations C3, C8 and C11. We report here the results obtained for an applied aspiration rate of 5 ml/s.

In absence of stroke the blood supply to the left side of the brain originates mostly from the left proximal ICA, with minor contributions from the PCoA. The largest part of this supply is directed to the perfusion of distal MCA territories, while the remainder supplies the proximal ACA and then the distal ACA. The introduction of an additional inlet, the aspiration catheter, severely affects this circulatory dynamics, causing retrograde flow along the retrieval path and its neighboring vessels.

When the aspiration is applied in the proximal ICA (configurations C3 and C8), the clot must be dragged through this vessel as well. In both cases, however, modifications in arterial flow during clot retrieval are negligible. In the first scenario, the mean ACA flow exhibits a distribution with median 360 ml/min (retrograde). Similar values are found in the proximal and distal ICA throughout the entire procedure. In the PCoA, which in healthy, well balanced conditions carries low blood volumes, flow is centered around 130 ml/min, while the flow in the Ophthalmic artery is close to zero. In all the vessels, the direction of flow is toward the aspiration point, signifying that advantageous haemodynamics conditions are created and the clot is retrieved under a favorable pressure gradient which opposes potential propagation of clot fragments toward the distal vasculature.

In case of aspiration located in the distal ICA (configuration C11), the retrieval path consists only of the MCA and the distal ICA itself. Flow in all vessels in this configuration is similar to the C3 and C8 cases. In this case, however, we observe lower flow in ICApp because of the combined effects of the distal position of the aspiration and the proximal position of the BGC.

The situation appears to be different in the MCA, where flow reversal is never achieved during surgery. Additionally, a more proximal location of the aspiration catheter induces a small increase in MCA flow.

Because of their qualitative similarity to the 5 ml/s case, results for 0.5 and 10 ml/s aspiration rates will not be discussed here in detail but are available in Supplementary material. The most notable difference is that, with 0.5 ml/s aspiration, half of the subjects show antegrade flow in ICAd with the aspiration located in the proximal part of proximal ICA. In the case of aspiration distal to the Ophthalmic artery, most of the patients present retrograde distal ICA flow, with a non-negligible minority (25%) having positive flow.

## 4. Discussion

In this study we presented a 1D computational framework for simulating the thrombectomy procedure with balloon guided catheter with occlusion in the MCA. The method was used to assess the influence of aspiration location and aspiration rate during clot retrieval. Results show the negligible effects of aspiration location on average MCA flow, which is instead more influenced by the applied aspiration rate. Net MCA retrograde flow was never achieved. Nevertheless, we showed that favorable haemodynamic conditions are obtained along the retrieval path and its neighboring vessels, even for low aspiration rates.

We coupled the 1D modeling approach with an ageing model in order to obtain a realistic baseline population of elderly individuals. This was particularly important because ageing modifies arterial properties such as their radius, important for simulating the retrieval of the clot, as well as their elasticity and peripheral boundary conditions, which affect the ability of the vessels to accommodate the variable flow regimes induced by the aspiration. Results in terms of brachial pressure are in excellent agreement with relevant *in vivo* (37) and *in silico* (4, 6, 41, 42) literature, and complex behavior such as the increase of diastolic pressure from 25 to 55 years, and its subsequent decline is successfully captured.

In terms of MCA velocity in healthy ageing individuals, our model predicts values of average velocity that are on the lower side of existing literature measurements (39, 40), particularly in younger populations. This is likely due to an underestimation of the cerebral peripheral resistance by the computational model. Similarly, we predicted values of MCA pulsatility index lower than those previously reported, possibly because of underestimation of vascular stiffness.

The importance of optimal positioning of the aspiration catheter during aspiration thrombectomy is well understood in the literature. The general consensus is that the aspiration tip should be deployed as close as possible to the thrombus, so as to minimize losses of aspiration force due to a non-complete engagement of the clot within the catheter (16). When this is not possible, deployment in the ICA is instead adopted. Our results show that the position of the aspiration catheter has only marginal effects on MCA flow, which remains antegrade in all twelve configurations. The main determinant to the flow is the externally applied aspiration, with stronger aspirations narrowing the MCA flow distributions and lowering their median values. This is consistent with existing observations that link greater aspiration forces to successful reperfusion and shorter procedural times (16).

In terms of MCA flow, we notice minor variations in MCA flow during retrieval for a given aspiration rate. Lowest values are attained when flow control is deployed proximal to the Ophthalmic artery. This is apparently in contrast with a previous study that observed better recanalization, but no statistically significant difference in long term clinical outcomes, in case of aspiration applied in the MCA (15). These results were obtained from retrospective analysis of a cohort of 101 patients who presented to hospital with MCA stroke. However, patients were classified in only two groups (distal versus proximal aspiration), and no further details were given on the position of the aspiration catheter within the proximal ICA. The patient cohort analysed by Baek et al. (15) was heterogeneous in terms of patients: some of them presented comorbidities such as diabetes and hypertension, known to induce modifications to the cardiovascular system (43), and the majority of them had already received intravenous thrombolytic drugs. Adverse effects such as aortic dissection and intracerebral hemorrhage, as well as higher mortality, were observed in the group treated with distal aspiration, leading the authors to conclude that proximal deployment is a valid alternative.

If the flow conditions in neighboring vessels is instead taken as an endpoint, analysis of the haemodynamic patterns shows that flows along the critical path are more favorable to the retrieval when using distal rather proximal aspirations, which supports previous findings that the aspiration catheter should be deployed as close as possible to the occlusion (10, 15). Our values of retrograde ICA flow, with median ranging from 200 ml/min (aspiration proximal to the Ophthalmic artery) to 250 ml/min (aspiration distal to the Ophthalmic artery) are well within flows measured by Okada et al. (44) in their synthetic model of the CoW (210 ml/min, retrograde). Contrary to us, they did observe reverse MCA flow of 100 ml/min. Mild reverse MCA flow was observed also in Chueh et al. (45), where they used a simplified *in vitro* representation of the cerebral circulation consisting only of ICA, PCoA and MCA. These discrepancies may possibly be ascribed to inconsistent boundary conditions between the experimental data and our simulations. Additionally, differences in network topologies may explain the different outcomes. In our model, the procedure fails in inducing retrograde MCA flow because of the flow contribution from the Anterior Communicating Artery and the ACA, which constitute a connection between the damaged brain side and the healthy, contralateral one. In agreement with this observation, a recent study used a 3D printed phantom of the entire CoW, proximal ICA and basilar artery, and reported values of antegrade flow of 40 ml/min during a simulated aspiration thrombectomy (46). Retrograde MCA flow was observed only when large bore aspiration catheters were used in the MCA. A precise comparison with our prediction of average values of 90 ml/min in the MCA with aspiration in the MCA, is not possible because the authors report an aspiration pressure of 700 mmHg but not the aspiration flow rate.

### 4.1. Limitations

The model we presented has some limitations. First, 1D modeling relies on the assumptions of straight arteries and doesn’t capture the geometrical complexity of vessels such as the ICA, whose tortuosity has been proposed as a predictor of the outcome of MT procedure (47, 48). Clinical reports (14) and studies from Bridio et al. (49) have confirmed this, and have shown that for some configurations it is not possible to successfully perform the retrieval.

We did not include an explicit representation of the catheters and did not modify the radius of catheterized arteries. Catheterized arteries can be approximated as two concentric tubes where the blood is only allowed to flow in the cavity between the walls (17). This configuration presents higher resistance to flow and, in pressure driven systems such as the cerebral circulation during aspiration thrombectomy, can hinder blood circulation and reduce the efficacy of the aspiration procedure.

We modeled the obstruction to flow caused by the clot/thrombus in two different ways: complete flow obstruction, represented through full wave reflection boundary conditions when the clot is located in the MCA at the beginning of the procedure, and as a partial occlusion of the vessels crossed during the retrieval process. The mechanical properties of the thrombus are known to influence its fragmentation during retrieval and the post-surgery outcome, as has been proven experimentally (10) and, recently, in a validated 3D fluid dynamics model of aspiration thrombectomy (24). Clot fragmentation cannot be directly enforced within our framework, but reduced order models based on principal component analysis of FE results (50) combined with an estimate of the pressure gradient at the extremities of the clot (51), provide a way to evaluate the load acting on the clot using haemodynamic variables derived from 1D simulations.

It is known that physiological compensatory mechanisms are in place during IS which alter the status of the distal circulation and consequently the pressure gradients during retrieval. Examples of such mechanisms are the collateral flow due to the leptomeningeal anastomoses (LMAs), an extensive network of small arterioles which connect different parts of the brain and guarantee continuity of perfusion following occlusion of large vessels such as the MCA (52), and autoregulation mechanisms which modify peripheral resistance and compliance. LMAs were not included in this model as previous studies (27–30, 52, 53) have shown that they act in restoring perfusion in distal brain districts and have little effect on blood flow in the CoW and the retrieval path. Similarly, autoregulation intervenes to support perfusion in infarcted tissues and there is no conclusive evidence that it induces modification in large proximal vessels (54).

Finally, we recognize that the conclusions drawn from our results on the optimal location of the aspiration catheter are based on purely fluid dynamics considerations, whereas in clinical settings doctors tend to evaluate procedural outcome through post-thrombectomy perfusion (TICI, thrombolysis in cerebral infarction), as well as use of the mRS (modified Rankin Scale) and NIHSS score (National Institutes of Health Stroke Scale) for long term neurological assessment of the patients. The aim of our work, however, was to show that the developed framework is able to effectively and realistically simulate a thrombectomy procedure, and to use it for a preliminary evaluation of the optimal deployment of the aspiration catheter.

In conclusion, we have developed a computational framework for simulating mechanical thrombectomy in ageing populations. We used it to characterize the optimal location for the aspiration catheter in case it can not be deployed proximal to the occlusion. The results support the conclusions that balloon guided catheters induce favorable haemodynamic conditions. The adoption of distal aspirations, when possible, should be preferred. However, proximal aspirations are still able to sustain the retrieval of clot while keeping a low risk of distal embolization, and represent a valid alternative.

## Data availability statement

The original contributions presented in this study are included in the article/Supplementary material, further inquiries can be directed to the corresponding author.

## Author contributions

IB: conceptualization, data curation, formal analysis, investigation, methodology, software, validation, visualization, writing–original draft, writing, and review and editing. AMu: conceptualization, methodology, visualization, writing, and review and editing. NW: methodology, writing, and review and editing. APN: conceptualization, methodology, supervision, writing, and review and editing. AN and AMa: funding acquisition, supervision, writing, and review and editing. All authors gave approval for the final version of the manuscript to be published.
