# Supplementary material for: A multiscale computational framework to evaluate flow alterations during mechanical thrombectomy for treatment of ischaemic stroke
Source: Front Cardiovasc Med. 2023 Mar 15;10:1117449. doi: 10.3389/fcvm.2023.1117449 (PMC10050705; doi:10.3389/fcvm.2023.1117449)
Supplement: Supplementary file 1 [file Table_1.DOCX]

Supplementary Material

A multiscale computational framework to evaluate flow alterations during mechanical thrombectomy for treatment of ischaemic stroke

Ivan Benemerito*, Ahmed Mustafa, Ana Paula Narata, Andrew Narracott, Alberto Marzo

*** Correspondence:** Ivan Benemerito: i.benemerito@sheffield.ac.uk

# Supplementary Data

Table 1: Network parameters for the baseline model at 25 years.

| Artery | *l*  (cm) | *R_0_*  (cm) | *E*  (kPa) | *R_1_+R_2_*  (10^9^ Pa·s·m^-3^) | *Cp*  (10^-10^ m^3^·Pa^-1^) |
| --- | --- | --- | --- | --- | --- |
| Ascending aorta | 4 | 1.13 | 384 | - | - |
| Aortic arch 1 | 2 | 1.02 | 317 | - | - |
| Brachiocephalic | 3.4 | 0.65 | 380 | - | - |
| Aortic arch 2 | 3.9 | 1.04 | 297 | - | - |
| Common carotid L | 20.8 | 0.25 | 375 | - | - |
| Common carotid R | 17.7 | 0.27 | 429 | - | - |
| Subclavian R | 3.4 | 0.40 | 403 | - | - |
| Thoracic aorta | 15.6 | 1.01 | 390 | 0.35 | 52.31 |
| Subclavian L | 3.4 | 0.40 | 403 | - | - |
| Ext carotid L | 17.7 | 0.14 | 976 | 3.85 | 1.23 |
| Prox Int carotid 1 L | 14.4 | 0.23 | 890 | - | - |
| Prox Int carotid 1 R | 14.4 | 0.23 | 890 | - | - |
| Ext carotid R | 17.7 | 0.14 | 976 | 3.85 | 1.23 |
| Vertebral R | 14.8 | 0.15 | 749 | - | - |
| Brachial R | 42.2 | 0.40 | 379 | 19.15 | 1.54 |
| Brachial L | 42.2 | 0.40 | 379 | 19.15 | 1.54 |
| Vertebral L | 14.8 | 0.15 | 749 | - | - |
| Dist Int carotid L | 0.5 | 0.18 | 1794 | - | - |
| PCoA L | 1.5 | 0.07 | 1895 | - | - |
| PCoA R | 1.5 | 0.07 | 1895 | - | - |
| Dist Int carotid R | 0.5 | 0.18 | 1794 | - | - |
| Basilar | 2.9 | 0.15 | 1565 | - | - |
| MCA L | 11.9 | 0.14 | 1259 | 2.47 | 1.15 |
| MCA R | 11.9 | 0.14 | 1259 | 2.47 | 1.15 |
| ACA1 L | 1.2 | 0.12 | 1648 | - | - |
| ACA1 R | 1.2 | 0.12 | 1648 | - | - |
| PCA1 L | 0.5 | 0.09 | 1767 | - | - |
| PCA1 R | 0.5 | 0.09 | 1767 | - | - |
| ACA2 L | 10.3 | 0.12 | 1318 | 9.1 | 0.35 |
| ACA2 R | 10.3 | 0.12 | 1318 | 9.1 | 0.35 |
| ACoA | 0.3 | 0.07 | 1766 | - | - |
| PCA2 L | 8.6 | 0.10 | 1694 | 11.55 | 0.41 |
| PCA2 R | 8.6 | 0.10 | 1694 | 11.55 | 0.41 |
| Ophthalmic L | 4.1 | 0.04 | 659 | 27.26 | 0.19 |
| Ophthalmic R | 4.1 | 0.04 | 659 | 27.26 | 0.19 |
| Prox Int Carotid 2 L | 3.3 | 0.23 | 890 | - | - |
| Prox Int Carotid 2 R | 3.3 | 0.23 | 890 | - | - |
